# Arteriovenous Malformations—Current Understanding of the Pathogenesis with Implications for Treatment

**DOI:** 10.3390/ijms22169037

**Published:** 2021-08-21

**Authors:** Katharina Schimmel, Md Khadem Ali, Serena Y. Tan, Joyce Teng, Huy M. Do, Gary K. Steinberg, David A. Stevenson, Edda Spiekerkoetter

**Affiliations:** 1 Division Pulmonary, Allergy and Critical Care Medicine, Department of Medicine, Stanford University, Stanford, CA 94305, USA; schimmka@stanford.edu (K.S.); mdali@stanford.edu (M.K.A.); 2 Vera Moulton Wall Center for Pulmonary Vascular Disease, Stanford University, Stanford, CA 94305, USA; 3 Department of Pathology, Stanford University, Stanford, CA 94305, USA; serenayt@stanford.edu; 4 Department of Dermatology, Lucile Packard Children’s Hospital, Stanford University, Stanford, CA 94305, USA; jteng3@stanford.edu; 5 Department of Radiology (Neuroimaging and Neurointervention), Stanford University, Stanford, CA 94305, USA; huymdo@stanford.edu; 6 Department of Neurosurgery and Stanford Stroke Center, Stanford University, Stanford, CA 94305, USA; cerebral@stanford.edu; 7 Department of Pediatrics, Division of Medical Genetics, Stanford University, Stanford, CA 94305, USA; dasteven@stanford.edu

**Keywords:** vascular anomalies, arteriovenous malformations, hereditary hemorrhagic, telangiectasia, endothelial cell, pathogenesis, clinical trials, repurposed drugs

## Abstract

Arteriovenous malformations are a vascular anomaly typically present at birth, characterized by an abnormal connection between an artery and a vein (bypassing the capillaries). These high flow lesions can vary in size and location. Therapeutic approaches are limited, and AVMs can cause significant morbidity and mortality. Here, we describe our current understanding of the pathogenesis of arteriovenous malformations based on preclinical and clinical findings. We discuss past and present accomplishments and challenges in the field and identify research gaps that need to be filled for the successful development of therapeutic strategies in the future.

## 1. Introduction

Arteriovenous malformations (AVMs) are abnormal shunts between arteries and veins that bypass the capillary bed. Complications arising from these direct arteriovenous communications are dependent on their anatomical location and stage of development and include stroke, brain abscess, hypoxemia, or local rupture with hemorrhages that can be life-threatening [1,2,3]. Current treatment modalities focus mainly on surgical, catheter-guided interventions or stereotactic radiosurgery aimed at resecting, embolizing, or radiating AVMs to manage the associated risks [3,4,5,6]. However, reperfusion due to recanalization, previously unrecognized feeding, or adjacent arteries leads to recurrence of AVMs in about 25% of patients within the first-year post-intervention [7,8]. Additional medical treatments for multiple or diffuse small AVMs are highly sought after, for which surgical interventions are not considered safe, technically feasible, or effective. 

The lack of FDA approved medical treatments for AVMs reflects our limited understanding of the pathogenesis of AVMs, in particular how AVMs develop and progress. Mounting evidence suggests that even for familial AVMs, underlying genetic defects in the germline alone might not be sufficient for AVMs to manifest. Local events such as somatic mutations and pro-angiogenic stimuli might be additionally required drivers [9,10,11,12,13]. Insights from experimental animal models employing cell-specific deletions of causative genes suggest the endothelial cell (EC) as the key cell type in AVM formation [14,15,16,17,18]. For the successful development of new therapies, identification of either one or multiple key pathways responsible for a change in EC behavior would be of high importance. Over the last decades, it has become increasingly clear that causative mutations, either somatic or germline, in a single AVM are causing gene results in a complex interplay between multiple cellular pathways [19,20,21]. Here, we review past and current observations from both clinicians and researchers that have led to emerging hypotheses for AVM formation. We also highlight the genetics, natural history, diagnosis, histopathology, preclinical model systems, and altered pathways, as well as promising medical approaches targeting relevant pathways for AVMs.

## 2. Genetics of AVMs

Although many AVMs are isolated, they can also be associated with a number of syndromes. A classic example of a syndrome associated with AVMs is hereditary hemorrhagic telangiectasia (HHT). Clinical diagnostic criteria for HHT were developed [22], now known as the Curaçao criteria, where solid organ AVMs are one of the diagnostic criteria. The solid organ AVMs in HHT typically occur in the brain, lungs, and liver (although they can also be present in the spine). HHT is an autosomal dominant condition with an incidence estimated at 1:10,000 [23]. The majority of individuals fulfilling the clinical diagnostic criteria for HHT harbor pathogenic variants in four known genes involved in the transforming growth factor-beta pathway [i.e., Endoglin (*ENG)*, Activin Receptor Like 1 (*ACVRL1* or *ALK1*), SMAD family member 4 (*SMAD4*), Growth Differentiation Factor 2 (*GDF2* or *BMP9*)] [24]. Loss of function mutations in either *ALK1* (HHT type 1) or *ENG* (HHT type 2) are the most common and occur in over 85% HHT cases, while the other two gene mutations are relatively rare. For example, *SMAD4* (Juvenile polyposis/HHT3) accounts for only 2% of cases and *BMP9* (HHT4) for less than 1% cases [24].

Another genetic disorder with AVMs is capillary malformation-arteriovenous malformation (CM-AVM) syndrome. CM-AVM syndrome is classically characterized by multifocal oval capillary malformations with a surrounding white halo and AVMs. A report by Wooderchak-Donahue [25] suggested that care providers had difficulty differentiating between HHT and CM-AVM syndrome likely reflecting the overlapping features such as AVMs. The AVMs in CM-AVM syndrome typically are reported in the brain, spine, muscle, bone and skin [26]. It is inherited in an autosomal dominant fashion due to loss of function variants in two genes [RAS P21 Protein Activator 1 (*RASA1)* and Ephrin Receptor B4 (*EPHB4*)]. Molecular testing on a biopsy of a capillary malformation on an individual with CM-AVM syndrome showed a somatic variant in addition to the germline variant suggesting a role for a “second hit” for the development of a vascular lesion in CM-AVM syndrome [27]. 

Phosphatase and Tensin Homolog *(PTEN)* hamartoma tumor syndrome (PHTS) has variable expressivity but can also have AVMs. It is an autosomal dominant syndrome due to pathogenic variants in the *PTEN* gene and is a tumor predisposition syndrome. Features classically associated with *PTEN* hamartoma tumor syndrome include autism spectrum disorder, macrocephaly, and benign and malignant tumors (e.g., breast, thyroid, endometrial, kidney, colon) [28]. Although vascular malformations may not frequently be thought to be a cardinal sign of *PTEN* hamartoma tumor syndrome, vascular malformations are not rare in this condition. At one center, 54% of individuals with germline *PTEN* pathogenic variants had a vascular malformation, and of those, 86% had fast-flow vascular anomalies such as AVMs [29]. The vascular malformations seen in PHTS typically were not found in the intrathoracic or intra-abdominal viscera, rather primarily in the extremities [29].

The genetic etiologies of non-syndromic isolated sporadic AVMs have not been well elucidated. It is likely that somatic variants contribute to their development and is an area ripe for investigation. One hypothesis is that sporadic AVMs could be due to somatic changes in genes associated with already known germline genetic syndromes with AVMs. There are limitations in identifying the genetic variants in such situations given concerns of obtaining the correct tissues for testing when the pathogenic events are attributed to genetic changes of low allele frequency. One group was able to identify somatic pathogenic variants in *MAP2K1* in extracranial AVMs [30,31]. This is intriguing for potential therapeutic repurposing approaches given the availability of MEK1 inhibitors already utilized for other indications. Further work to delineate genetic variants associated with sporadic AVMs could help in developing personalized therapeutic approaches.

## 3. Natural History of Different AVM and Their Clinical Characteristics

Non-syndromic AVMs often present as solitary lesions involving the extremities, pelvis, and head/neck. Literature about the natural history of AVMs is limited and most of the publications to date have focused on head and neck AVMs [32]. AVMs progress throughout life and cause complications including tissue destruction due to rapid overgrowth, bleeding, functional deficits, severe deformities, and high-output cardiac failure. 

Approximately 50% of extracranial AVMs are noticeable at birth. They grow with the child and about 80% will become noticeable by puberty. Nearly all of the extracranial AVMs progress and are diagnosed by adulthood with a slight predilection in females. During childhood, AVMs can present as an asymptomatic pulsatile mass associated with increased asymmetrical overgrowth of the affected tissue and overlying capillary stains. Evolution occurs at a variable rate, although it is well-known that progression is stimulated by a number of events such as trauma, puberty, and pregnancy. It is not uncommon for affected women to report notable expansion during pregnancy [32,33]. Over time, shunting increases, causing localized venous hypertension, reducing perfusion pressure to the involved and adjacent tissues that result in tissue ischemia with pain and subsequent tissue ulceration with bleeding. Extensive extracranial AVMs, and those with large fistulas cause cardiac volume overload, potentially leading to high output cardiac failure. Bony involvement as the primary site may occurs more often in the head and neck area, especially in the maxilla and mandible. 

Schobinger’s clinical staging is frequently used to categorize the risks and severity of AVMs [34,35]. Stage I is known as quiescence or inactive phase with localized hyperthermia. Patients, especially children with small AVM can remain stable in stage I for long periods of time [36]. Expansion (stage II) with an increase of arterio-venous shunting is often noticed due to increased pulsation and bruit, and is usually followed by pain, bleeding, as well as localized tissue destruction or ulceration (stage III). Once these symptoms and signs of progression occur within a large AVM, patients are often at risk of cardiac involvement and decompensation (stage IV).

## 4. Hypotheses of AVM Formation

To develop novel effective therapeutic strategies for AVM treatment, a better understanding of the pathogenesis of AVM formation is paramount. Hypotheses on how AVMs form and evolve are still a matter of debate and are based on clinical observations employing different imaging modalities as well as on identification of molecular, genetic, and cellular key-player in affected tissues of humans and experimental animals.

In 1990, a systematic study of biopsied cutaneous telangiectatic lesions of HHT patients was conducted using a combination of light and electron microscopy [37]. The lesions ranged in size from pinpoint to 2 mm. They were 3D-reconstructed from serial ultra-thin sections and analyzed for changes in the vascular bed to generate an overview of the development of AVMs. A sequence of distinctive morphologic events was reported. First, there was dilatation of postcapillary venules, surrounded by a mononuclear infiltrate mainly consisting of lymphocytes. Next, luminal diameter and vascular wall thickness of venules increased, accompanied by recruitment of pericytes. Enlarging and convoluting venules connected to arterioles, which also enlarged, through capillary segments. Finally, these segments disappeared, meaning the capillary bed had been completely replaced by direct arteriolar-venular connections. A possible explanation for this phenomenon might be found in studies showing that increased flow in a microvascular bed can cause capillaries to transition to arterioles [38,39]. Of note, ECs of both arterioles and venules connecting with each other in the fully developed AVM showed no ultra-structural abnormalities [37]. However, the shape of the tortuous and dilated venules was abnormal. In addition, there was an abrupt disappearance of elastic fibers at the arteriolar end and an excess in smooth muscle layers, as well as stress fibers in pericytes along venules. Collectively, these studies suggest that formation of a cutaneous AVM in HHT emerges around a postcapillary venule and is composed of three defined stages (dilation of postcapillary venules, formation of connections between arterioles and venules, and disappearance of capillary bed), ultimately leading to a direct communication between an artery and a vein.

The three steps of AVM development identified above were also described in patients with pulmonary AVMs (PAVMs) in HHT using high resolution CT scanning [11]. The initial stage of AVM formation presented as a ground glass nodule on a CT scan. It corresponded to the early telangiectasia of postcapillary venules and the inflammatory cell infiltrate. Subsequently, small vessels became visible within the ground glass opacity on CT. This was reflective of the venous branching towards enlarging arterioles. A fully matured PAVM was seen as a feeding pulmonary artery, an enlarged draining vein, and an aneurysmal sac like structure in between.

In familial AVMs, the question of why AVMs arise in seemingly random anatomic locations in an individual carrying the allele with the causative germline gene mutation within every cell has been a conundrum. A three-event hypothesis suggesting concurrence of two additional, regionally confined triggers, is mainly based on insights gained from animal studies. First, mice that were heterozygous in either one of the HHT causing genes, presented very mild HHT-like features, at a penetrance below 50% [40,41,42]. This suggested that loss of one functional allele in HHT-genes is not enough for the development of AVMs. One of the additional triggers proposed was a local complete loss of protein, or loss of heterozygosity. Mice with blepharitis that were heterozygous in the HHT genes endoglin (*ENG*) had more pronounced hemorrhagic lesions in the eyelids [42]. In an inflammatory environment, the extracellular domain of endoglin is cleaved off and released to bind to circulating ligands [43]. This causes a transient absence of endoglin in ECs. Of note, patients with sporadic cerebral AVMs had increased levels of the soluble form of endoglin in their circulation [44]. Local loss of protein either through shedding as described above, or additional somatic mutations [13] was therefore coined as a second contributor. The third event thought to trigger AVM development is an angiogenic stimulus. Dermal wounding or local application of angiogenesis stimulating agents such as Vascular Endothelial Growth Factor (VEGF) or Lipopolysaccharide (LPS) were required for AVM formation in adult inducible knockout mice that were homozygous for a deletion in HHT genes [10,14,15,17]. Even a complete loss of protein alone was not sufficient for AVMs to grow in these mice. In summary, these studies suggested that a combination of a loss of one functional allele in an HHT-locus, a local loss of protein, and an angiogenic stimulus directed towards ECs might be required for AVM development in mice.

Furthermore, determinants such as blood flow and shear stress have been identified to be contributory to AVM formation. Healthy ECs migrate against the direction of flow in vitro. In contrast, ECs migrate in the same direction as the flow upon loss of endoglin. This could be recapitulated in vivo, where loss of endoglin led to a reduced migration against the blood flow and a subsequent accumulation of ECs on the venous side of capillaries [20]. This supports the notion that the formation of AVMs in HHT might originate at the venous side.

Further research will be required to strengthen our understanding of AVM formation, and translate these insights from animal models to humans.

## 5. Overview of the Histopathology of AVMs, Vascular-Nonvascular Cells

AVMs are one of four types of malformations described in the current International Society for the Study of Vascular anomalies (ISSVA)-approved classification of vascular anomalies, with the other three including capillary malformations (CMs), lymphatic malformations and venous malformations [45]. When present on their own, they are known as “simple” malformations. When comprised of a mixture of two or more “simple” malformations, they are known as “combined” malformations.

The histologic appearance of AVMs is highly variable and range from simple AVMs most often seen sporadically and in isolation, to more complex lesions with features that overlap with simple AVMs, but with additional distinctive characteristics.

Simple AVMs are typically composed of larger caliber elastic arteries and thick-walled veins that are often admixed with beds of arterioles, venules and capillaries as well as stromal tissue. Although the actual connections or shunts between the arterial and venous component are not always readily apparent, evidence of the effects of the arteriovenous shunting is usually discernible. In well-developed lesions with higher grade shunts, the arteries are often tortuous and variable in caliber, with fragmentation and thickening of the elastic laminae. The veins, on the other hand, tend to show intimal hyperplasia and thickening of the perivascular smooth muscle and adventitial fibrosis due to the high-pressured and turbulent arterial flow. 

Syndromes that feature AVMs include CM-AVM syndrome associated with *RASA1* [26,46] or *EPHB4* [47] pathogenic variants, HHT, and syndromes associated with prominent overgrowth (e.g., *PIK3CA*-related overgrowth syndromes).

The histologic characteristics of the cutaneous vascular lesions associated with the CM-AVM syndrome have not been thoroughly documented and has been described in just a few studies. Although dilated, thin-walled dermal vessels are uniformly observed, these are thought to be distinct from typical CMs. One study suggests that these superficial vessels may represent changes secondary to a deeper AVM [48], and another study describes a prominent arteriolar component in these lesions, more akin to AVMs [49]. 

**Figure 1:**
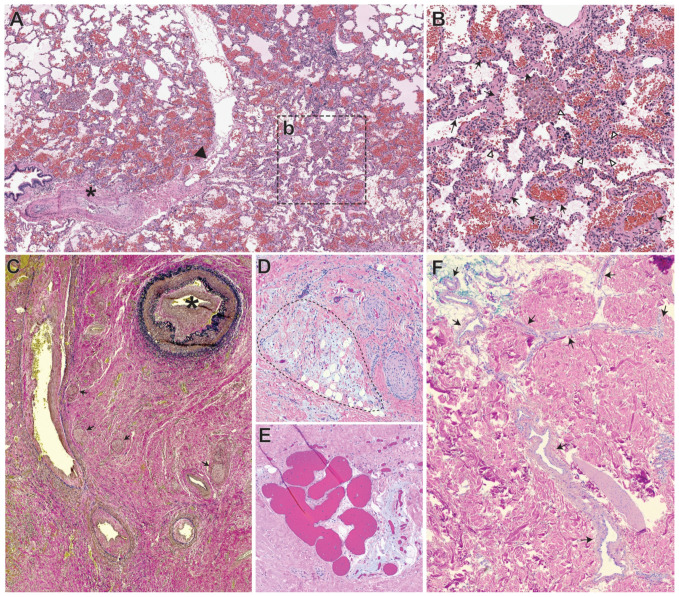
Examples of arteriovenous malformations (AVMs). (**A**,**B**) Arteriovenous malformation in the lung from a patient with hereditary hemorrhagic telangiectasia (HHT), *ENG* mutation, as well as pulmonary arterial hypertension (PAH), involving an artery with neointima (*) joining an interlobular septum containing pulmonary veins (arrowhead) (**A**). Adjacent to this fistula is a tangle of arterioles and venules (inset b is magnified in (**B**)) embedded within the alveolar parenchyma. These small vessels (arrows) often have smooth muscle in their walls, distinguishing them from alveolar septae (white arrowheads) (**C**) Elastin van Gieson stain highlights haphazardly distributed elastic arteries in a PTEN-mutated hamartomatous vascular malformation. The largest artery in the top right (*) demonstrates evidence of turbulent flow including thickening of the elastic laminae, prominent intimal hyperplasia and narrowing of the lumen. Neural hyperplasia (arrows), seen in the background, is a frequent finding in PTEN-mutated lesions. (**D**,**E**) Striking foci of myxoid change (delineated with dashed line) (**D**) and clusters of thin-walled vessels with a honeycomb-like arrangement (**E**) are additional characteristic features of PTEN-mutated lesions in addition to the arteriovenous component. These foci (**D**,**E**) are from a PIK3CA-mutated vascular malformation, which can show overlapping features with PTEN-mutated lesions. (**F**) Sporadic cutaneous AVM with a MAP2K1 variant, showing a web of arterioles and venules (arrows). Note that the changes in the vascular wall are subtle, suggesting lower grade shunting.

The spectrum of vascular lesions in HHT include mucocutaneous telangiectasias as well as visceral AVMs involving the lung, liver, brain, and gastrointestinal tract. Although AVMs in the setting of HHT are often multiple in number, they are histomorphologically similar to sporadic AVMs (Figure 1A,B).

The vascular lesions associated with PHTS often contain a high-flow arteriovenous component and are associated with other distinctive features that have been more extensively characterized [50]. They are composed predominantly of a visually bizarre mix of adipose tissue, fibrous to fibromyxoid stroma, with scattered clusters of thin-walled cavernous channels, venules and veins with variably-thickened smooth muscle coats, and haphazard bundles of smooth muscle and hypertrophic nerves. The arterial component is distinctive, often with abnormal elastic lamellation (Figure 1C), variable caliber and markedly narrowed lumina. Similar lesions have been described in PROS [51] (Figure 1D,E).

Finally, sporadic non-syndromic AVMs, which have been found to contain mutations affecting the MAP kinase pathway, for example *MAP2K1* [30] or more infrequently *KRAS* [52], often have the histologic appearance of a simple AVM (Figure 1F). 

Interestingly, mutations in *MAP2K1* and *KRAS* have recently been described in several intramuscular hemangioma capillary type (IHCT) fast-flow lesions [53]. Histopathologically, these lesions contain a prominent lobular capillarous component with admixed arteries and veins that exhibit changes reflective of anomalous vascular flow. Although the relationship of IHCT to typical AVMs remain ill-defined, their overlapping features suggest that they may lie on and perhaps expand the spectrum of AVMs.

## 6. Diagnosis of AVMs

### 6.1. Extracranial AVMs

Sporadic extracranial AVMs are solitary and may be localized or regional. Characteristic clinical features of AVMs include a palpable thrill approximal to the affected site with overlying red or purple skin discoloration and soft tissue overgrowth. AVMs can be painful on palpation when tissue ischemia starts to occur. Superficial AVMs (i.e., lip, external auditory canal, etc.), or AVMs involving the mucosa, can also present with a history of significant bleeding. Intracranial AVMs are often diagnosed following hemorrhage or neurologic manifestations. 

Multiple radiologic modalities have been utilized to assist in the diagnosis of AVMs [54], in addition to provide information on extension, composition and diameter of the feeding and draining vessels that are essential for therapeutic interventions. For extracranial AVMs, rapid blood flow can be demonstrable by doppler ultrasonography, which shows multiple hypoechoic channels with low-resistance arterial waveform throughout with no associated soft-tissue mass. Magnetic resonance imaging (MRI) will reveal signal voids consistent with fast-flow. MRI with time-resolved gradient echo will show a rapidly enhancing nidus, where abnormal arteries connect with draining veins [55,56]. The lack of an enhancing soft tissue mass is an important feature distinguishing AVMs from vascular tumors. For the extremities, draining veins are often asymmetrically enlarged in comparison to the contralateral draining vein. 

Dynamic contrast-enhanced MR angiography is also used to improve visualization of flow dynamics, inflow arteries and outflow veins and the location of shunting (nidus). Time-resolved 4D-CT-angiography (4D CTA) with low tube voltage settings can provide a new dimension of ultrafast dynamic imaging. For pediatric patients, reduction of the radiation dose and contrast media should be considered. However, catheter angiography remains a prerequisite for treatment of AVMs [56,57].

To diagnose pulmonary AVMs, a transthoracic contrast echocardiography (TTCE) to screen for the presence of a right-to-left shunt secondary to PAVMs should be performed [58]. In case of a moderate or severe shunt (Grade 2 or 3) on TTCE, a subsequent chest CT-scan will visualize the pulmonary AVM, the number and size of the feeding arteries, the nidus of the AVM as well as the draining vein allowing for planning of embolization procedures. [4,59].

It is recommended that screening for liver VMs be offered to adults with definite or suspected HHT. Diagnostic assessment of liver VMs with symptoms and/or signs suggestive of complicated liver VMs (including heart failure, pulmonary hypertension, abnormal cardiac biomarkers, abnormal liver function tests, abdominal pain, portal hypertension, or encephalopathy) can be performed with various imaging modalities such as Doppler ultrasound, multiphase contrast CT scan, or contrast abdominal MRI [4]. 

### 6.2. Intracranial AVMs

Cerebral AVMs can occur sporadically or as part of a genetic syndrome. The overall prevalence rate of cerebral AVMs in HHT patients is 10% with no significant differences in male versus female patients and pediatric versus adult patients [60]. Patients with an *ENG* mutation (HHT1) have a nearly seven-fold prevalence rate (13.4%) as compared to patients with an *ACVLR1* mutation (HHT2) (2.4%). Overall, 42.2% of HHT patients with intracranial AVMs have multiple brain AVMs. Nearly 20% of HHT cerebral AVM patients presented with hemorrhage and over half had symptoms attributable to their brain AVMs. Over 80% of AVMs were Spetzler-Martin grade of 2 or less, supratentorial in location and smaller than 3 cm; most AVMs were small or micro-AVMs in size [60]. The similar rates of brain AVMs in pediatric and adult HHT patients suggest that de novo formation of brain AVM in this population is low [61,62,63]. Most experts agree that patients with HHT should be screened for cerebral AVM at least once in adulthood. Given that even large cerebral AVMs can occur in childhood, children with HHT or being at risk for HHT (having a parent with HHT) should be screened at time of presentation/diagnosis [4]. This is supported by a high proportion of patients who suffer from symptoms related to their AVM and/or rupture. The imaging modality of choice to detect brain AVMs is brain MRI and magnetic resonance angiography (MRA). Besides routine MR sequences, the addition of arterial spin labeling, contrast, and 4D-time resolved MRA sequences increase the accuracy of MR [64,65]. Cerebral angiography is still considered more sensitive and the gold standard imaging modality for the detection of brain AVMs, but is increasingly being replaced by MRI. Angiography is performed when treatment or treatment planning is needed. Brain AVMs can be safely obliterated with multiple modalities: Microneurosurgery, neuroendovascular embolization, radiosurgery or a combination of these techniques [3,66,67,68]. Pretreatment imaging with stereotactic MRI and 3D rotational angiography aid in the localization of the AVMs both for surgery and radiosurgery. MRI with diffusion tensor imaging is helpful in surgical and radio surgical planning in order to avoid injury to eloquent brain structures [69].

Since somatic mutations have been identified in over 50–80% of intracranial and extracranial AVMs, tissue biopsy paired with peripheral blood can be used increasingly to confirm a diagnosis when clinical and radiologic features are not characteristic. Obtaining tissue from fast-flow vascular malformation however is not without risk especially at some anatomical locations. Therefore, there have been growing interests in the development of using cell free DNA for genetic diagnostic testing. Zanner et al. [70] demonstrated recently that genetic variants can be detected in plasma in two out of eight patients with extracranial AVMs. However, the challenge of low detection power remains due to low allele frequency that is often associated with some somatic disorders. It is also questionable whether this technology will be applicable to intracranial AVMs where risk of biopsy is probably the highest and non-invasive diagnostic tools are highly sought after. 

## 7. Overview of Model Systems of AVMs In Vivo (Zebrafish, Mouse Mutants, Antibody Treatment)

Studies recapitulating characteristic clinical features of AVMs in animal models have started to unravel causal factors of AVM pathology and to some extent informed clinical care. Advantages and disadvantages of each animal model are discussed in great details elsewhere [71,72], we here focus on important insights the community has gained from experimental animal studies.

### 7.1. Genetic Models

Germline genetic syndromes associated with AVMs were replicated by deleting genes known to be accompanied with the respective inherited syndrome, mainly in mice and zebrafish. Resulting data have significantly enhanced our understanding of basic functions of relevant proteins, and their importance for specific cell types.

In initial studies, total loss of HHT genes *ENG* (*ENG*^−*/*−^) or *ALK1* (*ALK1*^−*/*−^) resulted in embryonic lethality in mice [40,73,74,75]. Both *ENG*^−*/*−^ and *ALK1*^−*/*−^ yolk sacs contained enlarged, fragile vessels. *ENG*^−*/*−^ embryos had defects in the cardiac cushion and a delay in the maturation of major vessels with less vascular smooth muscle cells (vSMC) around them. Similar to that, *ALK1*^−*/*−^ embryos showed delayed vessel maturation. In conclusion, both proteins seem to be critical for angiogenesis, and endoglin additionally for cardiac cushion formation. Of note, one study reported large arteriovenous connections in *ALK1*^−*/*−^ embryos with reduced vSMC coverage and lower levels of genes important for arterial vessel identity [76]. This observation gave rise to the thought that the formation of AVMs in HHT during development might be due to a loss of identity in arteries and veins.

Subsequently, mice with one functional and one deleted allele in either one of the above gene loci were generated [40,41,42]. Resulting heterozygous *ENG^+/^*^−^ and *ALK1^+/^*^−^ mice developed HHT-like vascular lesions between seven and twenty months, and three and twenty-four months, respectively. However, these studies reveal that lesions occurred in less than half of the animals, and in an unpredictable fashion. Interestingly, lesions were both more frequent and more prominent in the 129/Ola background, implying a potential relevance for genetic modifiers in susceptibility to HHT [40,42]. 

To produce a model that better reflects the HHT phenotype in postnatal life, *ENG-* and *ALK1-*inducible knockout mice were generated [10,14,15,16,17,18,77,78,79]. As a matter of fact, some of the time-dependent and cell-type-specific homozygous deletions achieved in these animals succeeded in reproducing AVMs that resemble the ones of patients. Importantly, these studies identified the EC as the critical cell type in AVM development in HHT. Inducible deletion of either *ENG* or *ALK1* demonstrated that loss of *ENG* or *ALK1* in ECs, but no other cell types such as vSMCs, pericytes or macrophages, leads to formation of AVMs. Furthermore, it became clear that in adult animals, in which developmental pro-angiogenic signals are absent, other angiogenic triggers or inflammation were required for AVMs formation in the skin or the brain in addition to *ENG-* or *ALK1* deficiency. Further research is required to determine to which extent these findings translate to the condition of a human patient.

*ENG* or *ALK1* have also been successfully deleted in zebrafish, in which the endothelium can easily be visualized using fluorescent proteins [80,81,82]. Zebrafish models of cerebral AVMs are particularly useful because their entire cranial circulation can be seen in vivo, and systemic hemodynamic responses occurring in humans are mimicked, such as high output cardiac failure [82]. 

Testing of potential drugs for AVM treatment in genetic animal models has just begun to gain momentum. The ablation of relevant genes in mice or zebrafish will be a valuable resource for pre-clinical evaluation of improved therapeutic strategies for patients with AVMs in the future.

### 7.2. Antibody Based Models

In contrast to genetic models, where one specific gene can be deleted in either one or both alleles, antibodies can block an entire pathway relevant for familial AVMs. This was achieved recently by transmammary-delivered immunoblocking of *BMP9* and BMP10, the endothelial-cell-specific ligands of the BMPR2/TGF-β pathway, which harbors the genes that are mutated in HHT (i.e., *ENG*, *ACVRL1/ALK1,* or *SMAD4*) [83]. Lactating female mice were injected intraperitoneally with anti-*BMP9*/10 antibodies, which could be detected in the circulation of the offspring and induced AVMs in the retina. Follow-up research used this model to show protective effects of tacrolimus, rapamycin and nintedanib on AVMs in various anatomic locations [84,85]. Further studies are required to assess the feasibility of such therapy in humans.

### 7.3. Surgical Models

Surgical animal models have been helpful particularly in refining endovascular and radiosurgical techniques used in the clinic [71]. Radiosurgery primarily has two applications in this context.

**First**, it can be used as a treatment option in which a high-dose radiation beam focused on an AVM will lead to its obliteration [86,87]. While exact mechanisms are still unknown, local DNA damage induced by the beam triggers endothelial and smooth muscle cell proliferation, and ultimately thrombosis [88,89,90]. Avoiding AVM angiogenesis after the intervention is of utmost importance to prevent recurrence of the AVM. AVM angiogenesis post-radiosurgery or post-gamma knife surgery is highly dependent on the dose of the radiation beam. This was demonstrated in a study that implanted resected AVMs from humans into the corneal tissue of rats [91].

**Second**, radiosurgery might be used as a priming technique for vascular targeting. Vascular targeting has been successful to induce thrombosis in the field of cancer, where molecular differences between tumor and normal vessels can be used as a vascular target. To test whether radiosurgery might induce molecular changes that could serve as vascular targets, a carotid jugular anastomosis rat model was used. As a matter of fact, thrombosis was successfully induced in this animal model following radiosurgery by radiation induced expression of endothelial adhesion molecules such as E-selectin [92,93].

Another field of study that heavily relies on surgical animal models is AVM induced altered hemodynamics. Questions related to venous hypertension, hypoperfusion, thrombosis or chronic ischemia in or around AVMs can be addressed. For example, gamma knife surgery on AVMs in rats after surgical anastomosis of the left external jugular vein to the side of the common carotid artery led to significantly lower blood flow through both venous and arterial sides of the shunt [71].

Collectively, surgical animal models have been valuable to refine interventional techniques currently used in the clinics to manage AVMs.

## 8. Pathways and Crosstalk between Pathways

As discussed above, loss of function mutations in either *ENG*, *ACVRL1/ALK1*, *SMAD4*, or *BMP9* are associated with AVM formation and HHT. Interestingly, proteins encoded for these genes belong to the member of TGFβ superfamily and are all involved in the same signaling pathway [94]. The ligands *BMP9*/10 are present in the blood and bind with high affinity to a cell surface receptor complex consisting of a type I receptor such as *ALK1*, and a BMP type II receptor (BMPRII or ACTRIIA or ACTRIIB) and the coreceptor *ENG*. Noteworthy, loss of function mutations in BMPR2 can cause familial PAH, a different rare pulmonary vascular disease seen in some patients with HHT [95], suggesting the functional interaction between BMPR2 and *ALK1*. In ECs under healthy conditions, circulatory *BMP9*/10 activates the cell surface receptor complex, phosphorylates the transcription factors SMAD1/5/8, which binds to co-SMADs (e.g., *SMAD4*), translocates to the nucleus where it binds to a BMP response element DNA sequence (BRE) and acts as transcriptional regulator of target gene expression. Several non-canonical BMP signaling pathways are also activated by *BMP9*/10, including p38 Mitogen-Activated Protein Kinase (MAPK), Extracellular Signal-Regulated Kinase (ERK), Wingless (Wnt) and NOTCH signaling [96]. Dysregulation of BMP signaling have been shown to be associated with vasculature malformation diseases such as HHT [97]. In patients with HHT, impaired BMP signaling in ECs leads to a pro-angiogenic phenotype [96,98]. Previous studies also showed defective *BMP9* signaling in HHT causing mutants [99,100,101]. Furthermore, animal models of HHT in which *ENG*, *ALK1*, or *SMAD4* were inactivated, show vascular defects such as AVMs and hypervascularization [15,16,17,18,80,102]. Yet, the exact signaling pathway and downstream targets that contribute to the development of AVM and HHT pathogenesis are not fully understood [72]. Several studies suggest that HHT is linked to aberrant reactivation of angiogenesis, and the overactivation of pro-angiogenic pathways such as VEGF signaling, contributing to the development of the vascular pathology in models of HHT [12,103]. VEGF is a potent pro-angiogenic factor widely recognized as a major player in diseases associated with vascular malformations. In ECs, VEGF binds to VEGFR2 and stimulates proliferation, migration, tube formation, and thus promotes vascular remodeling and angiogenesis. VEGF levels have been shown to be increased in the resected brain AVM tissue (bAVM) [104,105,106,107,108], and in the plasma and skin telangiectasias of HHT patients [109,110,111]. Abnormal microvessels were found in the brain of *ENG* heterozygous mice following treatment with human recombinant VEGF156 [112] and in *ENG* conditional knockout mice (EC and SMC) with virally overexpressed VEGF [14]. This suggests that VEGF stimulation may play a key role in bAVM formations under *ENG* insufficiency condition. Researchers have also found that virally overexpressed VEGF levels enhanced mortality and bAVM hemorrhage in *ALK1*-deficient mice [113]. Shao et al. demonstrated that VEGF expression is stimulated by ALK5 (and SMAD2 via activation of ALK5) and inhibited via *ALK1* (and SMAD1 via activation of *ALK1*) [114]. Thus, any mutation along the *ALK1* pathway (*BMP9*, *ACVRL1*, *ENG*, *SMAD4*) results in increased VEGF via a decreased *ALK1* signaling pathway. Blocking VEGF by VEGF neutralizing antibodies has been shown to prevent AVMs in *ALK1*-deficient mice. Although the exact mechanism by which the loss of function of *ALK1* and endoglin activates VEGF signaling has not been established, it has become clear that VEGF/VEGFR2 signaling is a critical part of the pathogenic process of HHT and a promising therapeutic target. VEGF blockade with bevacizumab is used already clinically to reduced epistaxis and gastrointestinal bleeding in HHT [10,115]. In addition to VEGF, *ALK1* shows cross-talk with Notch, angiopoietin 2 and Hippo signaling [96]. 

Furthermore, increased activation of PI3K/AKT signaling has been shown to be implicated in the development of AVM in HHT patients [21] and in mice models of HHT [19,20]. Phosphorylation of ribosomal protein S6, a downstream component of mTORC1 and Akt, has been shown to be elevated in the ECs of AVMs in the *ALK1*-deficient mouse retina and in *BMP9*- and BMP10-immunoblocked mice [19]. Moreover, pharmacological inhibition of PI3K with wortmannin, a nonselective PI3K inhibitor, partly decreased the number of AVMs on the retinas of cell specific inducible (i) mouse models such as *ENG*iEC, *ALK1*iEC, and *SMAD4*iEC mice [19,20,116]. In addition, loss of Akt1 prevented AVM formation in *SMAD4*iEC mice [116]. A recent study by Ruiz et al., showed that combined treatment with sirolimus, an mTOR inhibitor and nintedanib, a receptor tyrosine kinase, corrected endothelial Smad1/5/8, VEGFR2 and mTOR signaling and thereby opposed signaling characteristic of HHT pathogenesis [85]. This suggests that a combined approach of correcting these signaling pathways could be effective in treating HHT. 

RAS/MAPK signaling plays a critical role in cell proliferation, growth, survival, senescence, and development [117]. Germline or inherited mutations activating the Ras-MAPK pathway are linked with diseases such as CM-AVM. In addition, using next generation sequencing technology, recently somatic mutations were identified in genes in the RAS-MAPK pathway in sporadic cases of AVMs and extra-neural AVMs [30,118]. This suggests a potential signaling link between syndrome-associated and sporadic cases of AVM development. As *KRAS* activating mutations were found in human bAVM lesions, the researchers further investigated the downstream signaling pathways in EC-enriched cultures derived from those bAVMs. They found that the MAPK-ERK and PI3K-AKT pathways were activated by *KRAS* activating mutations. There was an increased level of ERK1/2 phosphorylation in ECs isolated from bAVMs compared to cells isolated from normal brain vessels. The mutations were shown to increase expression of genes related to angiogenesis and Notch signaling. Remarkably, even the VEGF gene signature was reversed in ECs with inhibition of MAPK-ERK suggesting that inhibition of the MAPK pathway might be a promising target for certain brain AVMs by targeting multiple pathways simultaneously [52,118].

## 9. Targeting Vascular Malformations with Repurposed Drugs; Benefits of Combinational Therapy (Surgical/Medical) for Complex AVM

AVMs are challenging vascular malformations to manage. Surgical debulking and embolization by interventional radiology have been the main stay of intervention for AVMs [8,119,120]. Even when surgical interventions are feasible, a complete cure, especially for AVMs involving the head, neck and intrathoracic area is rare, as surgical resection margins are usually difficult to determine. Subtotal excision often results in rapid recurrence and progression. Review of a large series of AVMs in 272 patients [8] showed an approximately 57% recurrence rate within one year after resection (with or without embolization) and an 86% recurrence with embolization alone within a year. The rapid recurrence following surgical intervention is attributed to possible neo-angiogenesis due to elevated levels of vascular endothelial growth factor and angiopoietin resulted from wound healing. To date, there is no FDA approved medical treatment for AVMs. Due to transient benefit with rapid disease relapse, surgical interventions are usually reserved for those AVMs with complications or end organ dysfunction. 

Recent advancement in understanding the pathogenesis of AVMs has uncovered the roles of somatic mutations such as *MAP2K1* (extracranial) and KRAS (intracranial) [30,69,118]. Inactivating mutations in RAS p21 protein activator 1 (*RASA1*) cause CM-AVM syndrome which is characterized by randomly distributed capillary malformations and predisposition for cerebral and noncerebral AVMs. *PTEN* mutations associated with *PTEN*-tumor hamartoma syndrome (PTHS) are also associated with increased high-flow vascular malformations, including AVMs [29,119]. Over the past decade, a number of targeted anti-angiogenic agents received regulatory approval for solid tumor and hematologic malignancies. The shared genetic variants in AVM and oncologic disorders suggest the potential use of targeted oncological therapies for complex vascular anomalies. Repurposing these therapies will likely expedite discovery of effective medical treatments for complex vascular anomalies including AVMs.

As described above, many recent studies have demonstrated that PIK3CA/ATK/mTOR and RAS/BRAF/MEK/ERK are two key cellular signaling pathways involved in the pathogenesis of AVMs. *RASA1* and *PTEN*, known to associate with syndromic AVMs, may also upregulate the mTOR pathways. Therefore, therapies inhibiting these two pathways will likely be beneficial for patients with complex AVMs. Sirolimus, everolimus and temsirolimus are well known inhibitors of the mTOR pathway that have been used for a number of tumorigenic disorders [120,121]. A Phase II study by Hammill et al. demonstrated safety and efficacy of sirolimus in the treatment of various vascular tumors and malformations [122]. In vivo animal models showed that sirolimus inhibits angiogenesis via downregulating the PI3K/AKT signaling pathway and the expression of VEGF. Although published studies to date have shown limited response of AVMs to sirolimus alone [122], it has been demonstrated as an effective adjuvant treatment when used in combination with sclerosing therapy for complex head and neck AVMs [123]. Patients treated with combined sirolimus and embolization have had a much more sustained response. As mentioned above, tacrolimus, sirolimus and nintedanib as combination therapy have been successfully tested in antibody based preclinical models of AVM formation and could represent a promising adjunct therapy for surgical AVM treatment [84,85]. Overexpression of MAP2K1 in ECs results in disruption of vascular channel formation, leading to overall abnormality of the capillary networks between developing arteries and veins in zebra fish [52]. Targeted therapies to BRAF and MAP2K1 are effective in inhibiting the formation of abnormal vascular network. Chelliah et al., have shown that a MEK inhibitor can be used to treat AVMs in an animal model [123]. A case report by Lekwuttikarn et al. demonstrated efficacy of trametinib in a ten-year-old girl with a rapidly progressive AVM [124,125]. The patient had failed eight months of continuous treatment with sirolimus prior. These preliminary observations suggest a genotype guided approach in the management of complex AVMs. There are multiple RAS/BRAF/MEK/ERK pathway inhibitors including vemurafenib, sorafenib, conimetinib that are currently on the market. Well-designed clinical trials are needed to evaluate the safety and efficacy of these targeted therapies.

## 10. Future Directions

In order to improve treatment and care of patients with AVMs, a plethora of research directions and disciplines need to work together like pieces of a puzzle, as illustrated in the graphical abstract: advances in understanding the underlying genetics, pathway alterations and pathobiology of AVMs using different preclinical models that reflect several aspects of the human disease have resulted in the identification of promising medical approaches. Multidisciplinary teams that utilize multi-modal approaches such as surgery, endovascular embolization, radiosurgery, medical therapy, are the key to translating the basic science findings into the clinic.

Further research is needed to understand the detailed mechanism of AVM formation and guide development of improved therapeutic strategies. Long standing hypotheses of AVM formation, such as the local occurrence of pathologically relevant second hit factors or the three-event hypothesis for AVM development in HHT, require thorough investigation. Efforts have been limited in the past by the accessibility of clinical samples harboring visceral AVMs with paired unaffected tissue. Such endeavors would be important however to clarify if therapeutic approaches should be directed towards increasing transcriptional levels of wildtype alleles, activating downstream signaling following a complete loss of function mechanism, or correction of cross talking pathways that drive the development of the disease.

Medical treatments that could prevent, halt or even reverse vessel remodeling in AVMs would be life changing in particular for children to prevent growth, complications and co-morbidities of surgically insufficiently treated or untreatable AVMs. Finally, pre-clinical models that more closely reflect human visceral AVMs as well as the study of human cells and human AVM tissue will likely lead to novel insights into molecular and cellular defects. This will enhance our understanding of AVM formation and enable new treatment options for these challenging clinical lesions. 

## Data Availability

Not applicable.
